# Endocrinology in the Time of COVID-19: A Rapid Evolution of Knowledge and Care

**DOI:** 10.3390/medicina57080805

**Published:** 2021-08-06

**Authors:** Ali A. Rizvi, Anca Pantea Stoian, Nader Lessan, Manfredi Rizzo

**Affiliations:** 1 Division of Endocrinology, Metabolism, and Lipids, Department of Medicine, Emory University School of Medicine, Atlanta, GA 30322, USA; ali.abbas.rizvi@emory.edu; 2 Division of Endocrinology, Diabetes and Metabolism, Department of Medicine, University of South Carolina School of Medicine Columbia, Columbia, SC 29208, USA; manfredi.rizzo@unipa.it; 3 Diabetes, Nutrition and Metabolic Diseases Department, Faculty of General Medicine, Carol Davila University of Medicine and Pharmacy, 050474 Bucharest, Romania; 4 The Research Institute, Imperial College London Diabetes Centre, Abu Dhabi, United Arab Emirates; nlessan@icldc.ae; 5 Department of Health Promotion, Mother and Child Care, Internal Medicine and Medical Specialties (Promise), University of Palermo, 90133 Palermo, Italy

American singer-writer and visual artist Bob Dylan produced the song “The Times They Are a-Changin” in the 1960s, which became a rallying cry for the civil rights and anti-war movements in that decade [1]. In a unique but analogous manner, the global community in modern times is facing a similar challenge of unprecedented proportions at the interface of health care, society, freedoms, and human existence as we have known it [2]. The onslaught of the SARS-CoV-2 infection, also known as COVID-19, has introduced rapidly evolving aspects to the understanding and management of the whole spectrum of medical information available to humankind. Indeed, the landscape has changed so radically that many concepts of care and disability that were once accepted as immutable have now been turned upside down [3]. 

Among the medical subspecialties that have been affected in a unique manner by COVID-19 has been the discipline of endocrinology. It is well-known that the treatment of diabetes forms the basic bread-and-butter of most clinical endocrinologists. The COVID-19 pandemic has affected multifaceted aspects of endocrine care. It is now well established that patients with diabetes are at a vastly higher risk of contracting more severe forms of the disease [4,5]; in fact, the relationship appears to be bidirectional [6]. Individuals with diabetes are at higher risk of life-threatening complications, mainly of a cardiopulmonary and renal nature [7,8]. Obese persons with previously existing pre-diabetes or mild diabetes, when exposed to the SARS-CoV-2 virus, develop a greatly advanced form of hyperglycemia that frequently requires life-sustaining care and hospitalization [9]. Moreover, apparently new-onset variations of the disease seem to have emerged that point to the possibility that the virus has a diabetogenic strain [10]. The treatment regimens for patients with advanced COVID-19 infections, such as high-dose corticosteroids, are obviously implicated in the aggravation of hyperglycemia. 

In the midst of the different aspects of diabetes care, the pandemic has vastly accelerated our propensity to employ all our so-called big guns up-front that are at our disposal. Situations that were historically conducive to a slow and methodical approach, perhaps influenced by therapeutic inertia, now require intensive treatment regimens and continuous glucose monitoring. In addition, combination therapies are gaining popularity [11]. The myriad of noninsulin agents for the management of diabetes that came to the horizon in the past few decades seem to have their own novel interplay with the COVID virus [12,13,14]. For example, both major groups of incretin-based therapies have their own set of benefits in concomitant COVID-19 infection, acting both via the glucagon-like peptide-1 receptors and independently of it [15]. The dipeptyl peptidase inhibitor-4 receptors are also a target for viral interaction though the exact significance of this finding is not fully understood [16,17]. Likewise, the sodium-glucose transporter-2 inhibitors are posited to have influence at the mechanistic level, which is still controversial [18]. 

Another huge jump of a logarithmic scale in the COVID era has been the galactic advances in health care technology covering two key aspects: glucose monitoring and telehealth delivery of health care [19]. The management of diabetes in the hospital setting received a boost with no objection from the FDA to the use of continuous glucose monitoring techniques; this step was initially taken to enhance the protection of health professionals caring for patients with known or suspected COVID-19 virus [20]. However, an unintended and rather salubrious consequence of this action was to utilize CGM for full therapeutic use, including initiation in the inpatient setting and transitioning to discharge and outpatient use [21]. Cloud-based and Bluetooth communication technologies enabled informed and motivated patients to communicate their glycemic profiles with their physicians on a regular basis, aided by portal-delineated electronic communication as a component of electronic medical records [22]. Complemented by rapid adoption of telemedicine use, this has changed the face of diabetes health delivery and brought high-quality care from the clinicians’ office to the patients’ doorsteps and living rooms [23]. The advantages in terms of convenience and safety are of such vast magnitude that it is fair to proclaim that telehealth is here to stay and grow.

Lest it be assumed that the influence of COVID-19 has been limited to diabetes, other endocrinologic health domains have not escaped the virus’s onslaught. Endocrine hypertension, with fluctuations in adrenal and renal hormone mechanisms, has gained a new understanding [24]. Pituitary function is exquisitely sensitive to inflammatory perturbations, and the pituitary-thyroid and pituitary-adrenal axes are prone to virus-induced derangements [25,26]. The thyroid gland, a key player in metabolism and homeostasis, can be affected by the SARS-Co-2 virus, manifesting functional changes and a thyroiditis-like picture [27,28]. Calcium and vitamin D metabolism and the parathyroid-gut-bone axis are also susceptible to changes induced by the virus [29,30]. Finally, yet importantly, adipose tissue and the release of cytokines of a deleterious, proinflammatory nature may be key players in the vascular endothelial response [31]. We should also consider gender difference in the battle against COVID-19 and the consequent impact of genetics, comorbidities, inflammation, and lifestyle on outcomes [32].

As well as direct effects on patients and patient care, the COVID-19 pandemic has affected endocrinology in other ways. Like other healthcare professionals, endocrinologists have had to learn and adapt to working in different and sometimes alien and difficult conditions with compulsory and necessary protective gear [33,34]. Even so, endocrinologists and their families have not been immune from COVID-19 infection, with sometimes serious consequences. Working days have been lost with obvious effects on patient care. Some have been affected by COVID infection nonetheless [35,36] and have experienced the potentially devastating effects of the virus on themselves and their families first hand [37]. This has also had effects on healthcare systems, which have had to cope with less than 100% of the workforce available at any one time. In financial terms, healthcare systems have been overwhelmed and stretched to limits not seen before [38,39,40], with negative effects on medical and nursing education and research [41]. 

It is critically important that a timely and updated compendium be put together of the vast amounts of knowledge and data related to endocrinology that has been gained since the beginning of the COVID-19 pandemic. With this lofty aim in mind, the editors of the special issue invite research papers and reviews pertaining to the unique and ever-evolving interface of COVID-19 and endocrinology. Following the lead of the great North American clinician and teacher Dr. William Osler, who declared that “medicine is a science of uncertainty and an art of probability,” we hope, with this effort, to bring some guidance and clarity to this ever-changing landscape. 
